# Clinical Characteristics of Juvenile Myasthenia Gravis in Southern China

**DOI:** 10.3389/fneur.2018.00077

**Published:** 2018-02-27

**Authors:** Xin Huang, Yingkai Li, Huiyu Feng, Pei Chen, Weibin Liu

**Affiliations:** ^1^Department of Neurology, The First Affiliated Hospital, Sun Yat-sen University, Guangzhou, China; ^2^Guangdong Provincial Key Laboratory for Diagnosis and Treatment of Major Neurological Diseases, Guangzhou, China

**Keywords:** juvenile myasthenia gravis, clinical characteristics, treatment, outcomes, southern China

## Abstract

**Objectives:**

To describe the clinical profile, clinical outcomes and factors that may affect the outcome of juvenile myasthenia gravis (JMG) patients in southern China.

**Methods:**

We reviewed information relating to JMG patients treated and evaluated at the First Affiliated Hospital, Sun Yat-sen University, between 1998 and 2015. The study involved 327 JMG patients who had been followed up for ≥1 year.

**Results:**

Overall, 77.4% patients showed initial symptoms in the prepubertal period (<12 years). 306 patients showed only ocular symptoms at onset. By the final follow-up, 61 ocular myasthenia gravis (OMG) patients (61/306, 19.9%) had developed generalized myasthenia gravis (GMG). Anti-acetylcholine receptor antibodies (AChR-Ab) titer was an independent risk factor for generalization. Eleven patients (3.4%) experienced spontaneous remission, but four relapsed. Low-dose oral prednisone (0.25 mg/kg) was administered when symptoms did not significantly improve after pyridostigmine treatment. Immunosuppressants were administered when prednisone was unsatisfactory. Optimal outcome was achieved in 59.6% of patients. Specifically, 60 patients (18.3%) attained complete stable remission (CSR), 12 (3.7%) attained pharmaceutical remission (PR), and 123 (37.6%) attained minimal manifestation (MM). In total, 53 OMG patients (21.5%) attained CSR, a significantly higher proportion than among the GMG patients (8.6%, *P* = 0.009). Moreover, 67.2% of patients with duration <2 years showed significant clinical improvement compared with 46.3% of those with duration >2 years (*P* < 0.001). Thymectomy did not exhibit definite efficacy for JMG patients.

**Conclusion:**

There was a low frequency of cases positive for AChR-Ab in the Chinese population. AChR-Ab titer was revealed as an independent risk factor for generalization. Low doses of prednisone could treat JMG effectively with few side effects.

## Introduction

Myasthenia gravis (MG) with onset in childhood or adolescence is termed juvenile myasthenia gravis (JMG) (1); the upper age limit for this condition is commonly set to 18 years of age (2). It is unclear whether the pathogenesis of JMG is the same as that of adults. Clearly, JMG and adult MG have many different characteristics, including symptoms, clinical severity, antibody titer, and thymus histology (3). The clinical features and demography of JMG patients are also significantly different when compared between different regions and ethnic groups. It is estimated that approximately 10–15% of MG cases in Caucasian populations involve juvenile onset (4). However, Asian studies have reported a relatively high incidence of JMG (2, 5). We previously reported that there is a high proportion of JMG patients, almost 50%, in the southern Chinese population (6). However, there have been few reports which have described the characteristics and long-term outcome of patients with JMG.

## Materials and Methods

Data were obtained from the First Affiliated Hospital Sun Yat-sen University MG Patient Database for patients seen in the hospital between 1998 and 2015. This database contains comprehensive demographic and clinical information for all in- and out-patients seen in the First Affiliated Hospital of Sun Yat-sen University. Patients were included if they had been diagnosed with acquired MG, not including congenital MG and neonate temporary MG, if the age of onset was less than 18 years and if patients had been treated in our hospital with a follow-up of at least 1 year. A total of 327 patients were enrolled for final analysis.

We collected a significant body of information: age of onset, gender, duration, presence and concentration of autoantibody, the Myasthenia Gravis Foundation of America (MGFA) clinical classification at maximum severity, and treatment modalities and outcome. Anti-acetylcholine receptor antibodies (AChR-Ab) were measured using an enzyme-linked immunosorbent assay (ELISA); an AChR-Ab titer >0.45 nmol/L was considered to be positive. The severity of the disease at onset was classified as type I, IIa, IIb, IIIa, IIIb, IVa, Ivb, or V according to published MGFA classifications (7).

Oral low-dose prednisone (0.25 mg/kg) was also employed when symptoms did not improve significantly after the administration of pyridostigmine. Prednisone was tapered using a specific schedule after symptoms had significantly improved and was eventually discontinued after symptoms had totally disappeared for at least 6 months. Immunosuppressants were administered when treatment efficacy was unsatisfactory, including cyclophosphamide, azathioprine, and leflumide. If the medication did not achieve the desired effect, thymectomy was considered. The change in clinical status was determined as: (1) Optimal: Minimal Manifestations (MM), pharmaceutical remission (PR), or Complete Stable Remission (CSR); (2) Intermediate: Improved; or (3) Unchanged and Worse (7).

The Student’s *t*-test, and the χ2 test were used to evaluate differences between groups, and a *P* < 0.05 was considered to be statistically significant. Variables which influenced treatment outcome were also evaluated by logistic regression analysis. All statistical analyses were performed using SPSS 19.0 (SPSS Inc. Chicago, IL, USA).

All patients provided informed written consent.

## Results

### Demographics

This study involved 327 JMG patients; Table 1 summarizes the clinical characteristics of these patients. The median age of these JMG patients at onset was 6 years. Patients were divided into the following categories according to age at onset: (1) infancy, less than 1 year; (2) early childhood, 1–3 years; (3) pre-school age, 3–6 years; (4) school age, 6–12 years; and (5) puberty, 12–18 years. The incidence of JMG in the infant group was the lowest; only nine patients (2.8%) were diagnosed within 1 year of birth. In contrast, 77.4% of patients with initial symptoms were classified into the prepubertal period (<12 years).

**Table 1:** Clinical characteristics of the 327 juvenile myasthenia gravis patients involved in this study.

Age at onset	Patients	Proportion (%)	Male	Proportion (%)	Female	Proportion (%)
<1 year	9	2.8	5	55.6	4	44.4
1–3 years	79	24.2	32	40.5	47	59.5
3–6 years	82	25.1	42	51.2	40	48.8
6–12 years	83	25.4	47	56.6	36	43.4
12–18 years	74	22.6	37	50.0	37	50.0

Total	327	100	163	49.8	164	50.2

Duration at first visit, months	44.9 ± 3.6 (0.1–360)					

There were 163 males (49.8%) and 164 females (50.2%), and the male:female ratio was1:1.01. The median age was 6 years and 5 years for males and females, respectively.

### Symptoms at Onset

In total, 306 patients (93.6%) showed only ocular symptoms at onset, including ptosis, diplopia, and/or strabismus. The most common initial presentation was ptosis, occurring in approximately 72.8% of patients (238/327).

Limb weakness occurred in 0.9% of patients (3/327) at onset, and only two patients (0.6%) presented with bulbar weakness at onset. One case presented with dysphagia, while another case presented with dysarthria. Two patients presented with respiratory muscle weakness at onset, and 14 (4.3%) presented weakness in two or more muscle groups at onset (Table 2).

**Table 2:** Symptoms at onset.

Symptoms at onset	Patients	Proportion (%)
**Ocular symptoms**		
Ptosis	238	72.8
Diplopia	16	4.9
Strabismus	7	2.1
Ptosis and diplopia	38	11.6
Ptosis and strabismus	7	2.1
Limb weakness	3	0.9
**Bulbar weakness**		
Dysphagia	1	0.3
Dysarthria	1	0.3
Respiratory muscle weakness	2	0.6
Ocular and limb weakness	7	2.1
Ocular and bulbar weakness	3	0.9
Ocular, limb and bulbar weakness	4	1.2

### Myasthenia Gravis Foundation of America (MGFA) Clinical Classification

**Figure 1:**
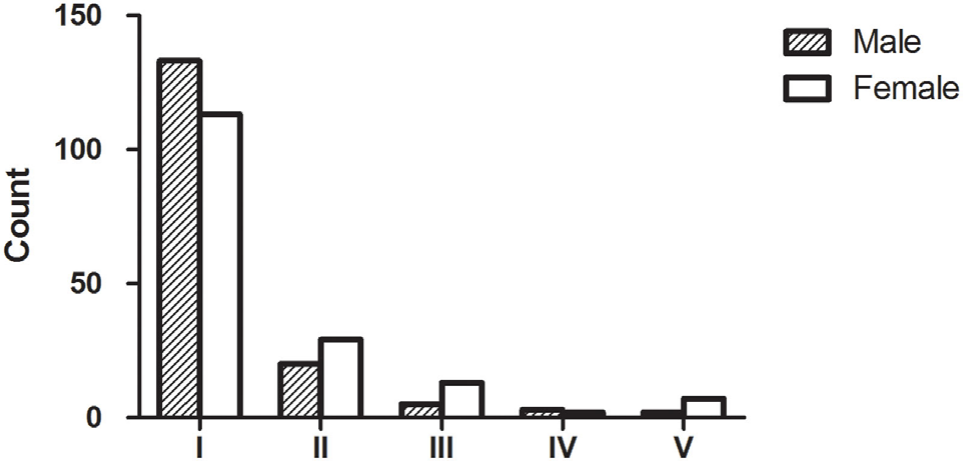
Myasthenia Gravis Foundation of America (MGFA) clinical classification and gender. In total, 327 patients with acquired myasthenia were classified according to the MGFA classification at maximum severity. Our data indicated that there were subgroups of MG patients that shared distinct clinical features or disease severity; most patients were MGFA class 1. The group distribution of MGFA classification was significantly different (Fisher’s exact test, *P* = 0.038) when compared between males and females.

All 327 patients were classified according to the MGFA classification at maximum severity. In total, 246 (75.2%) patients had ocular myasthenia gravis (OMG) and 81 (24.8%) had generalized myasthenia gravis (GMG). There were significant differences in MGFA classification between different genders (Fisher’s exact test, *P* = 0.038, Figure 1).

**Figure 2:**
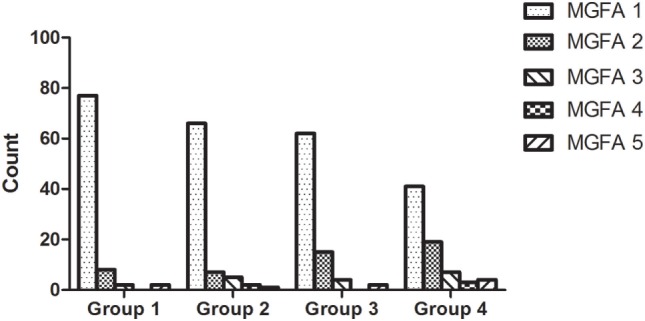
Myasthenia Gravis Foundation of America (MGFA) clinical classification across all age groups. Patients within different age groups had significantly different MGFA classifications (χ2 test, *P* = 0.004); there were more patients diagnosed as MGFA class 1 in the patient group which was <3 years of age. The proportion of MGFA class I patients decreased with age, while the proportion of MGFA class II patients increased with age.

Patients were divided into four groups, based upon their age (Age group 1: ≤3 years; Age group 2: 3–6 years; Age group 3: 6–12 years; Age group 4: 12–18 years). Patients within different age groups had different MGFA classifications (χ2 test, *P* = 0.004). Compared with other age groups, patients less than 3 years of age showed a statistically greater incidence of MGFA class 1 (χ2 test, *P* = 0.0003). In contrast, patients aged 12–18 years showed a significantly higher proportion of MGFA class 2 than other age groups (χ2 test, *P* = 0.003). There were no significant differences in the other three MGFA classifications when compared across the five age groups. The MGFA clinical classification across these five groups is shown in Figure 2.

### Serological Studies

Between 2007 and 2015, 185 patients were tested for anti-acetylcholine receptor antibodies (AChR-Ab; the test first became available in our hospital in 2007, so no data were available prior to this date). Of whom, 76.2% (141/185) were seropositive. Tests showed that 73.3% of the OMG patients were positive for AChR-Ab, and 84.0% of GMG patients were positive; this difference was not statistically significant (χ2 test, *P* = 0.13). Interestingly, 100% of the MGFA class 5 patients were positive for AChR-Ab. However, statistically, the positive rate of MGFA class 5 patients was not significantly higher than in the other groups (Fisher’s exact test, *P* = 0.736).

**Figure 3:**
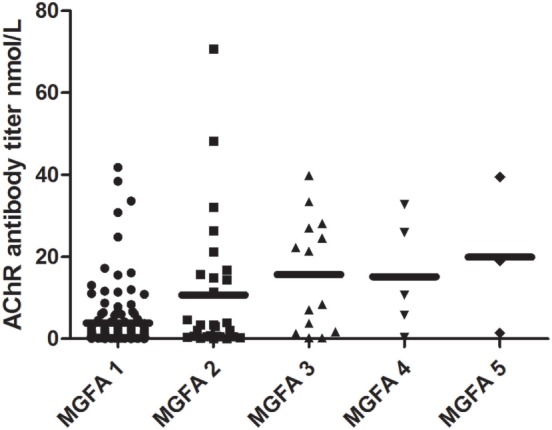
Distribution of AChR antibody titer across different Myasthenia Gravis Foundation of America (MGFA) classifications. Significant differences existed in AChR antibody titer when compared across MGFA classification groups (Kruskal–Wallis test, *P* = 0.002). AChR antibody titer was lowest in the ocular patients and highest in the MGFA class 5 patients.

There were clear differences in AChR-Ab titer across the different MGFA classification groups (Kruskal–Wallis test, *P* = 0.001). AChR-Ab titer was lowest in the ocular patients (3.80 ± 0.61 nmol/L) and highest in the MGFA class 5 patients (19.94 ± 10.98 nmol/L). The antibody titers of different MGFA classification groups are shown in Figure 3. The AChR-Ab titer of OMG patients (3.80 ± 0.61 nmol/L) was lower than that of GMG patients (13.07 ± 2.20 nmol/L; *P* < 0.001).

For each subject, we also tested other immune indicators, including humoral immune and cellular immune indicators. The rate of abnormal CD4 levels was 48.8%, while the rate of abnormal CD8 levels was 25.6%. However, mean CD4 and CD8 levels were within the normal range (32.9 ± 0.64%, 24.0 ± 0.50%, respectively). The means levels of IgG, IgM, and IgA were also within the normal range (10.55 ± 0.22, 1.42 ± 0.05, 1.55 ± 0.07 g/L, respectively). However, the rate of abnormal IgG levels was 60%, while the rate of abnormal IgM and IgA was 41.7 and 41.7%, respectively.

### Thymus Pathology

Within our study cohort, 184 patients received computed tomography (CT)/magnetic resonance imaging (MRI) in order to investigate thymic pathology (to insure the authenticity of the study, we verified the patients who had undergone thymus imaging studies and still possessed images, or copies of images). The most common histopathology was thymic hyperplasia and was detected in 164 cases (89.1%). Thymic atrophy was the rarest histopathology and was found in only three cases; 5 and 12 patients had thymoma and a normal thymus, respectively. The positive rate for AChR-Ab reached 100% in thymoma patients.

### Complications

Within our study cohort, 34 patients (10.4%) were found to have other autoimmune diseases; hyperthyroidism was the most common (31/34). Of these patients, 14 (45.2%) were females and 17 (54.8%) were males. Hyperthyroidism occurred before MG or at the same time as MG in 9.7% of patients (3/31), after MG in 48.4% (15/31), and the exact time was unknown in 41.9% of patients. Other autoimmune diseases included hypothyroidism (2/34) and nephrosis (1/34).

### Treatment and Outcomes

All patients were treated with pyridostigmine, except for spontaneous remission patients. Oral low-dose prednisone (0.25 mg/kg) was administered when symptoms did not improve significantly after the administration of pyridostigmine, and the dose was adjusted up or down, depending upon clinical response. Immunosuppressants were more commonly prescribed for cases that did not show a satisfactory response to pyridostigmine and prednisone. Thymectomy was performed in 226 patients (69.1%). Table 3 shows the treatment regime within each of the subgroups. The use of immunosuppressive agents in OMG patients was significantly lower than for GMG patients (χ2 test, *P* < 0.0001). There was no difference in terms of medical treatment between AChR-Ab positive and AChR-Ab negative patients (*P* = 0.069).

**Table 3:** Treatments in juvenile myasthenia gravis patients according to Myasthenia Gravis Foundation of America (MGFA) classification.

Treatment	MGFA I	MGFA II	MGFA III	MGFA IV	MGFA V
Pyridostigmine only	19(7.7%)	1(2.0%)	0(0%)	0(0%)	0(0%)
Pyridostigmine + Prednisone	109(44.3%)	13(26.5%)	5(27.8%)	0(0%)	4(44.4%)
Pyridostigmine + Immunosuppressants/Pyridostigmine + Prednisone + Immunosuppressants	117(47.6%)	35(71.4%)	13(72.2%)	5(100%)	5(55.6%)
Thymectomy	158(64.2%)	43(87.8%)	14(77.8%)	5(100%)	6(66.7%)

Optimal outcome was achieved in 59.6% patients, while 60 patients (18.3%) attained CSR. Fifty-three (21.5%) of the OMG patients attained CSR, significantly more than GMG patients (χ2 test, *P* = 0.009). In total, 12 patients (3.7%) attained PR: 10 of whom were OMG patients and 2 were GMG patients. Furthermore, 122 patients (37.3%) attained MM; of whom, 92 were OMG patients and 30 were GMG patients.

There was no difference in outcome between different genders (χ2 test, *P* = 0.33), and there were no significant differences in terms of outcome across the five different age groups (*P* = 0.728). However, there were differences in therapeutic effect across the different MGFA classification groups (χ2 test, *P* = 0.024).

Outcome was not significantly different between AChR-Ab positive and AChR-Ab negative patients and optimal outcome was achieved in 54.6% of AChR-Ab positive patients. Optimal outcome was achieved in 59.1% of AChR-Ab negative patients.

Significant clinical outcome depended upon the duration between onset and the administration of standard treatment. In the 67.2% patients in which the duration was less than 2 years, there was a significant clinical improvement as compared with the 46.3% of patients in which the duration was more than 2 years (*P* < 0.001).

Thymectomy did not show definitive efficacy in JMG patients. Whether patients were classified as being either OMG or GMG, the outcome was not significantly different when compared between those who were thymectomized and those who were not thymectomized (*P* = 0.83 and *P* = 0.759, respectively).

Univariate logistic regression analysis revealed that optimal outcome differed according to duration (*P* = 0.008) and MGFA classification (*P* = 0.02). As there were a small number of patients in MGFA class 3, 4, and 5, we combined these three classes into one group and referred to them as “severe MG patients.” This was to help minimize statistical errors and validate comparisons. Thus, we made comparisons between groups of MGFA class I patients, class II patients, and severe MG patients. However, multivariate logistic regression analysis revealed that duration and MGFA classification were irrelevant to treatment effect.

Eleven patients (3.4%) obtained spontaneous remission, although four patients relapsed.

### OMG Progress to GMG

306 patients showed only ocular symptoms at onset. By the time of our last follow-up session, 61 OMG patients (61/306, 19.9%) had transformed into GMG. The median age at onset was 8 years and the study population was dominated by females (1.9:1, F:M ratio). AChR-Ab were detected in 40 patients; 87.5% patients were positive. The mean AChR-Ab titer was 13.98 ± 0.16 nmol/L.

Univariate logistic regression analysis showed a significant risk of generalization for females (*P* < 0.001), age (*P* = 0.019) and AChR-Ab titer (*P* < 0.001). Multivariate logistic regression further revealed that AChR-Ab titer was an independent risk factor for generalization (*P* < 0.001).

Optimal outcome was achieved in 45.9% of patients showing generalization. After adjustment for gender and age at onset, the prognosis for generalization patients was worse than that for OMG patients (*P* = 0.019).

## Discussion

Previous studies in European and North American countries have reported that MG mainly affects adults (8–10). Furthermore, several studies have reported bimodal distributions with regards to the age of onset; for example, Thanvi and Lo reported a first peak to occur in the third decade and a second peak in the sixth and seventh decades (9). However, in China, JMG cases are estimated to account for more than half of the MG population (5). Our present study further indicated that in China, JMG has its own distinct characteristics.

We found that the age of onset of Chinese JMG is earlier than in other biogeographical regions. In our present study, most patients presented their initial symptoms during the prepubertal period (<12 years of age); we also identified that the median age at onset was 6 years. In Korea, the mean age at onset was reported to be 7.0 years (2). However, in Norway, the median age of onset was reported to be 13.0 years (1).

Juvenile myasthenia gravis shows no apparent gender bias. In our study, the male:female ratio was 1:1.01; this was lower than stated in our previous report on adult MG (6). However, our current data was similar to another study from China, which showed a male:female ratio of 1:1.01 (11). Some studies have reported an equal gender distribution in JMG patients with prepubertal onset (12, 13). Most studies revealed a higher incidence among females, especially in JMG patients with post-pubertal onset (2, 12, 14); consequently, it was considered that sex hormones might play an important role in the pathogenesis of MG. However, our present study did not identify such a pattern. Interestingly, male predominance has also been reported previously (15, 16). A Indian study, by Singhal et al., identified a male:female ratio of 2.70:1 (15). Collectively, this data indicate that the pathogenesis of MG is complex and include genetic predispositions, the environment, and a range of hormonal factors. Furthermore, direct comparisons between studies are difficult, because of different enrollment ages and clinical methodologies. Patients of different genders also have different peak ages at onset; the age of onset in females is earlier than males.

The present study showed that 75.2% of patients had OMG (MGFA class I). This compared to 86.3% of the JMG population in a Korean study in which patients presented with ocular symptoms (2). We believe that juvenile patients mostly present with purely ocular symptoms, particularly in Asian populations. An American study showed that only 35% of JMG patients had ocular symptoms (3). Thus, discrepancy among ethnicities appears to be very prominent. However, the proportion of patients with JMG with ocular symptoms in our current study was lower than that reported for the Chinese mainland population in a study by Gui et al. (5). These authors found that 95% of patients with childhood-onset MG showed ocular symptoms. The largest difference between this previous study and the current study is that the study population was different. We selected patients whose age of onset was less than 18 years while Gui et al. studied patients who were younger than 14 years. Coincidentally, we found that the proportion of MGFA class I patients decreased with increasing age.

Our data showed a lower positivity rate for AChR-Ab than other studies concerning JMG. Castro et al. reported that 84% of their patient group were AChR-Ab positive (3) while a Korean study showed a higher positive rate (2). This difference may be explained by the predominance of OMG in our current study. The AChR-Ab titer of OMG patients was less than that of GMG patients. Gui et al. reported a lower positive rate for antibodies because the proportion of OMG patients in the study population was higher (5). However, our research did not identify a clear correlation between antibody titer and prognosis.

Most studies have reported an increased proportion of CD4 cells in MG patients, but with a reduced proportion of CD8 cells and an increased CD4/CD8 ratio (17). Our study found that the proportion of abnormal CD4 and CD8 levels in peripheral blood was higher than normal. Therefore, we cannot infer whether MG patients exhibit hyper-functional or hypo-functional immune mechanisms. Consequently, we propose that there is an immune disorder associated with patients with MG.

Long-term high doses of prednisone have many side effects, such as growth failure and Cushing’s reaction. We used a low dose of prednisone therapy (0.25 mg/kg) to reduce the risk of such side effects. This form of treatment worked well, particularly for OMG. Immunosuppressants were prescribed for cases who did not show a satisfactory response to prednisone. Through long-term follow-up and with close monitoring, we believe that the use of immunosuppressive agents is both safe and effective.

Thymectomy is generally considered as an important option for treating MG patients. Castro et al. reported that JMG patients who underwent thymectomy showed a significant improvement in their symptoms (3) while Gui et al. proposed that thymectomy should be suggested for JMG patients with GMG. However, the present study did not provide a definitive conclusion for the use of thymectomy, either for OMG or for GMG patients. Some studies have indicated that thymectomy has not been demonstrated to prevent generalized progression and should therefore only be used in patients refractory to medical treatment (5, 18). Whether thymectomy is of benefit for patients, especially in JMG and OMG patients, remains the source of considerable debate. The study of “Randomized Trial of Thymectomy in Myasthenia Gravis (19)” showed the benefit of thymectomy. The guidelines of the international MG consensus also suggested that thymectomy could be used in JMG when pharmaceutical therapy does not meet its targets. Our present study showed that thymectomy was of no benefit for JMG, although this was a retrospective viewpoint. Furthermore, our goal was to be able to provide some evidence that could be sued to standardize the application of thymectomy in JMG.

We wanted to identify factors that could predict therapeutic effects. It appears that disease duration and MGFA classification may represent such factors. However, after adjusting our analyses for confounding factors, both duration and MGFA classification were shown to be irrelevant to the treatment effect.

The vast majority of our patients only showed ocular symptoms at onset. Long term follow-up revealed that some of these patients transformed into GMG; indeed, in the present study, 19.9% of patients with ocular presentation subsequently developed generalized symptoms. In a previous study, Gui et al. reported that 12.5% of patients with ocular presentation progressed to GMG (5). Furthermore, European and American studies revealed that up to 50% of patients progressed to GMG (20, 21). We suspect that genetic differences might be responsible for such differences between studies.

The chance of undergoing generalization was almost double in females compared with males. A previous study also found that female gender was a risk factor for generalization; this finding is consistent with several other studies (2, 22). The specific role of gender in the pathogenesis of MG, however, remains unclear. Some studies have suggested that females are more frequently affected by thymic hyperplasia and have higher antibody levels (7, 23, 24). Furthermore, our current data showed that patients with post-pubertal onset were more likely to generalize; these findings are also similar to previous research (3, 25) and confirm the influence of hormonal changes. Thus, we suggest that female patients with post-pubertal onset require active treatment, even if they present with only ocular symptoms at onset. Furthermore, patients with onset at infancy were also prone to progress to generalized myasthenia. The specific cause for this requires further exploration.

Anti-acetylcholine receptor antibody levels can represent an independent risk factor for generalization; with this respect, our opinion is consistent with that of Kupersmith et al. (26); Peeler et al. (27) also published similar results and related the higher rate of antibody sensitivity to the duration of symptoms.

Myasthenia gravis could be really difficult to differentiate from CMS with seronegative antibodies. We pay careful attention to differentiate diagnosis between MG and CMS, through the manifestation, antibody tests, EMG results, therapeutic diagnosis, and long time follow-up of the patients, especially seronegative patients with poor immunotherapy response. Fortunately, it’s still an ongoing follow-up study, new emerging available tests including special antibody test and gene test would solve this problem. Our totally follow-up duration was relative short, although most of the patients had been followed up for at least 2 years. The longer time (3 years or more follow-up) would provide insights into the long-term clinical outcome of patients.

## Conclusion

In conclusion, the data presented herein confirms the complexity of prognosis for JMG patients. We show that low doses of prednisone can treat JMG effectively with fewer side effects. We also demonstrate that AChR-Ab titer represents an independent risk factor for generalization. The strength of our study is that the data are population-based, with long-term follow-up by the same neurologists.

## Ethics Statement

This study was carried out in accordance with the recommendations of ethics committee of the first affiliated hospital of Sun Yat-sen University with written informed consent from all subjects. All subjects gave written informed consent in accordance with the Declaration of Helsinki. The protocol was approved by the ethics committee of the first affiliated hospital of Sun Yat-sen University.

## Author Contributions

XH participated in the diagnosis and treatment of most cases, contributed in data entry, statistical analysis, manuscript writing, and manuscript submission. YL contributed to database development and data processing, manuscript writing, and manuscript revision. HF participated in the treatment of some cases, contributed to data collection, and manuscript submission. PC contributed to data collection. WL diagnosed and treated most cases, established the database, designed, and supervised the experiment, data analysis, and manuscript writing.

## Conflict of Interest Statement

The authors declare that the research was conducted in the absence of any commercial or financial relationships that could be construed as a potential conflict of interest. The reviewers MM, JK and handling editor declared their shared affiliation.

## Abbreviations

JMG, juvenile myasthenia gravis; OMG, ocular myasthenia gravis; GMG, generalized myasthenia gravis; AChR-Ab, Anti-acetylcholine receptor antibodies; CSR, complete stable remission; PR, pharmaceutical remission; MM, minimal manifestation; MGFA, Myasthenia Gravis Foundation of America; ELISA, enzyme-linked immunosorbent assay.
